# Children’s first handwriting productions show a rhythmic structure

**DOI:** 10.1038/s41598-017-05105-6

**Published:** 2017-07-17

**Authors:** Elena Pagliarini, Lisa Scocchia, Mirta Vernice, Marina Zoppello, Umberto Balottin, Sana Bouamama, Maria Teresa Guasti, Natale Stucchi

**Affiliations:** 1 Center for Brain and Cognition (CBC), Departament de Tecnologies de la Informació i les Comunicacions (DTIC), Universitat Pompeu Fabra, c\ Ramon Trias Fargas, 25-27, Barcelona, 08005 Spain; 2 Department of Psychology, Università degli Studi di Milano-Bicocca, Piazza dell’Ateneo Nuovo, 1, 20126 Milan, Italy; 3 Child Neuropsychiatry Unit, C. Mondino National Neurological Institute, Via Mondino, 2, 27100 Pavia, Italy; 4 Centre for Visual Cognition, School of Psychology, University of Southampton, Building 44R 4049. University Road, SO17 1BJ Southampton, UK; 5 Department of Brain and Behavioral Sciences, University of Pavia, Pavia, Italy

## Abstract

Although much research has been concerned with the development of kinematic aspects of handwriting, little is known about the development along with age of two principles that govern its rhythmic organization: Homothety and Isochrony. Homothety states that the ratio between the durations of the single motor events composing a motor act remains invariant and independent from the total duration of the movement. Isochrony refers to the proportional relationship between the speed of movement execution and the length of its trajectory. The current study shows that children comply with both principles since their first grade of primary school. The precocious adherence to these principles suggests that an internal representation of the rhythm of handwriting is available before the age in which handwriting is performed automatically. Overall, these findings suggest that despite being a cultural acquisition, handwriting appears to be shaped by more general constraints on the timing planning of the movements.

## Introduction

Writing is one of the major cultural inventions by *Homo Sapiens* and it is a unique skill of our species. Since the beginning, the first scribes seem to have been aware that the shapes they chose should have been easy to write by the motor system and easy to recognize by the visual system. Indeed, it is not coincidence that underlying similarities common to all the world’s writing systems have been noticed^1–3^. The analysis of 115 writing systems from different classes (numerals, abjads, abugidas, alphabets and syllabaries, see Text S1) highlighted that all writing systems have an average number of about 3 strokes per character^1^. Characters are also *ca*. 50% redundant, meaning that the misrecognition of one or two strokes does not necessarily cause the misrecognition of the character^1^. Importantly, these traits appear to be independent of writing system size. Another major feature shared by all the world’s writing systems is that letters and symbols display regularities in their topological shapes and seem to match traits of objects recurring in natural scenes^2^.

Writing acquisition is also a major step in child development. Previous studies on handwriting have mainly focused on the development of different geometric and kinematic aspects, such as the length of the trace or the speed of execution^4–11^. This body of research showed that handwriting is performed automatically and ballistically from the third grade of primary school^4–6,8^. One aspect of handwriting which is often neglected is its rhythmic dimension, whereby rhythm is concerned with the durational features of events. Similarly to language and other human complex actions^12,13^, handwriting is not just a succession of isolated acts, but rather an organized, hierarchical process, in which the time and the space of each motor unit (i.e., strokes, letters, words) are contextually interdependent with each other within a larger unit^12–14^. As such, handwriting is governed by two principles of rhythmic organization: Homothety and Isochrony^12,15–22^.

Homothety rules the timing of the sub-components of the handwriting act (e.g., singular letter). According to Homothety^12,16,18–22^, the ratio between the durations of the single motor events that compose a motor act remains invariant and independent from the total duration of the movement. When applied to handwriting, Homothety predicts that the relative durations of the individual letters that compose a word are kept constant despite changes in the global duration, thus preserving their temporal relationships^19^. It appears clear that Homothety is nothing but the rhythm of handwriting. Apart from handwriting, a relative invariance in the rhythmic structure of the movement has been observed in typing^23,24^, gait^25^, and wrist movements^26^.

Isochrony^15,17,20^ governs the timing of the handwriting act as a whole (e.g., word). It states that the velocity of a movement is tuned to the length of the trajectory. Therefore, according to this principle, the speed of an intentional movement increases as a function of the linear extent of its trajectory, so as to keep the total duration of execution approximately constant. In regard to handwriting, Isochrony predicts that whatever the size of the handwriting, the duration of the execution of a movement is kept steady by increasing the writing velocity proportionally^19,21,27^. Besides handwriting, this compensatory mechanism has been observed in a variety of motor tasks such as drawing^21,28–31^, weight-lifting^32^, manual pointing^33^, voluntary contractions of human arm muscles^34^ and hand to target-object movement^35^. So, for instance, it has been shown that a more than ten-fold increase in size produces only a 50% extension of execution time when participants are asked to draw elliptical figures of different sizes^29^. Due to its substantiate properties, this empirical principle is now a well-established regularity in human movement and has recently been observed also in free-range macaques during reach-to-grasp movements^36^. In order to be satisfied, both principles require the coding of an abstract temporal internal representation before the beginning of a movement^31^. Therefore, handwriting is governed by general kinematic laws of human movement^20,21^ and two of them - Homothety and Isochrony - specifically rule the organization in time of its events.

The extraordinary evidence described so far suggests that writing is based on mechanisms inscribed in our biology despite being a cultural invention. At present, there is very little knowledge about what age children start to adhere to kinematic invariants when writing. To the best of our knowledge, no previous research to date has examined the development of Homothety, whereas the study of the development of Isochrony has been limited to scribbles and drawings, which are fairly simple spontaneous activities, mastered very early in life^28,31^. It is important to notice that Homothety and Isochrony are two independent principles, and therefore they might reveal a different developmental pattern. In one study^28^, 119 children ranging from 4;7 to 9;4 years of age were asked to draw circles of different sizes, first in an increasing order of size and second randomly. In another study^31^, 48 children ranging from a mean age of 5 years (SD 2;2) to a mean age of 11;11 years (SD 1;9) were given a set of 10 elliptic templates traced in an A3 paper in a random order and were asked to trace each ellipsis continuously for about 14 seconds. The results of these studies showed that children as young as 5 years of age adhere to Isochrony during scribbling and drawing activities. However, differently from scribbling and drawings, handwriting requires several years of training in order to become automatized and effortless, especially when writing in cursive script. Crucially, if handwriting has evolved within our brain’s constraint, we hypothesize that Homothethy and Isochrony do not require protracted training in order to be put in place, therefore these principles will characterize handwriting since the very first children’s production. In other words, we hypothesize that the very first handwriting productions of children will show a temporal structure with no developmental variation both when writing in all-capital block – the first script taught in first grade, and thus presumably the more automatized - and cursive script, which is presented later, and is usually less automatized with respect to all-capital block in the first years of school. This issue is particularly relevant if we consider very recent findings showing that Homothethy and Isochrony were consistently and substantially violated in handwriting by Children with Dyslexia and children with Dyslexia plus Dysgraphia of age 9, at variance with the results obtained for typically developing children who complied well with both principles^37^. Nevertheless, it is still unclear whether Homothety and Isochrony are inherent properties of handwriting or rather features that develop after some practice. Therefore, it is possible that Homothety and Isochrony are not respected by children with Dyslexia and children with Dyslexia plus Dysgraphia merely because they have less experience in handwriting than typically developing children.

In order to investigate whether Homothety and Isochrony are inherent properties of handwriting, we tested two hundred ninety-eight children from the first to the fifth grade of primary school in a handwriting task. Children were divided into five groups according to their school grade (a group of first grade children - henceforth G1; a group of second grade children - henceforth G2; a group of third grade children - henceforth G3; a group of fourth grade children - henceforth G4; and a group of fifth grade children - henceforth G5). Children were asked to write a target word (the Italian word *burle*; English translation “jokes”) under different manipulations of speed and size. Our task included the two extremes of each condition: *Big*/*Small* (size); *Fa*st/*Slow* (speed); *Spontaneou*s condition as baseline. Only Spontaneous, Big and Fast conditions were analyzed (*Materials and Method*). Children were asked to write the target word in the different conditions both in all-capital block and cursive scripts (Figure S1). Kinematics and trajectory of handwriting were collected on-line by means of a digitizing tablet. The duration of each single letter across different conditions was analyzed to investigate the adherence to Homothety. The relationship between the mean speed of movement when writing a word and the length of the word trajectory was analyzed in order to study the compliance with Isochrony.

## Results

All-capital block script and cursive script were analyzed separately due to structural differences between the two scripts. First, the two scripts are geometrically very different. Moreover, the handwriting of cursive script requires the connection of letters in a smooth and frictionless motion whereas the handwriting of block script in all capitals does not have smoothness requirements as each letter is written separately from the other, though it requires adequate word spacing. Furthermore, proficiency is likely to be different between these two scripts^37^. In the Italian educational system, the all-capital block script is introduced before the cursive script and is commonly practiced more often, especially in the first years of primary school. For these reasons, all the analyses from here on will be presented separately for the block script in all capitals and the cursive script.

Descriptive data are reported in Table S1. Preliminary analyses confirmed that children complied with the experimental requirements and modulated the speed and size of the handwriting according to the task conditions, both when writing in all-capital block script and in cursive script (Text S2).

### Homothety

#### Homothety: all-capital block script

The GLM analysis on the relative letter duration revealed a significant Group x Letter x Condition interaction, *F*(32, 2344) = 1.79, *p* < 0.01, η^2^_p_ = 0.02. For G1, the duration of the letters *b*, *u*, and *r* written in the *Big* condition were different with respect to those written in the *Fast* condition. Moreover, the duration of the letter *b* in the *Spontaneous* condition differed from that in the *Fast* condition. For G2, the duration of the letter *b* written in the *Spontaneous* condition differed from the *Big* and the *Fast* conditions. The duration of the letter *r* written in the *Spontaneous* condition differed from the *Fast* condition and the duration of the letter *r* in the *Big* condition differed from the *Fast* condition. For G3, the duration of the letter *b* written in the *Spontaneous* condition differed from that of the *Big* and *Fast* conditions. For G4, the duration of the letter *u* written in the *Big* condition differed from the *Fast* condition. No significant post-hoc comparisons were found for G5.

**Figure 1:**
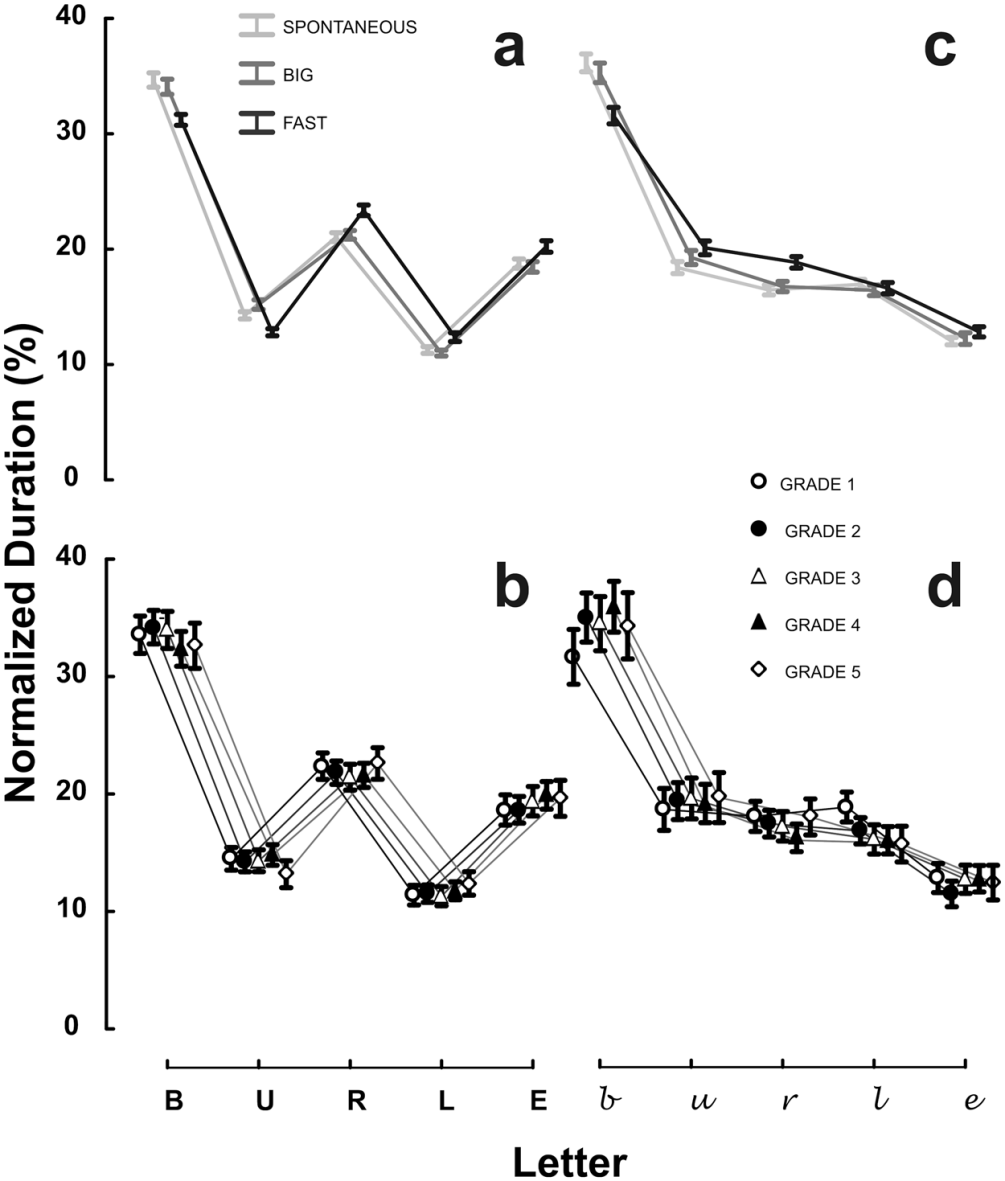
Homothety. The Condition (*Spontaneous*, *Big*, *Fast*) x Letter (*b*, *u*, *r*, *l*, *e*) (all-capital block script: Panel a; cursive: Panel c) and Group (G1, G2, G3, G4, G5) x Letter (*b*, *u*, *r*, *l*, *e*) (all-capital block script: Panel b; cursive: Panel d) interactions are shown for the percent duration taken to write each letter. Vertical error bars represent 95% confidence interval.

The analysis also revealed a significant Condition x Letter interaction, *F*(8, 2344) = 45.29, *p* < 0.001, η^2^_p_ = 0.13 (Fig. 1, Panel a). The duration of each letter in the *Spontaneous* condition differed from the *Fast* condition. The duration of each letter written in the *Big* condition differed from the *Fast* condition. No significant difference was found between the *Spontaneous* and the *Big* condition. The Group x Letter interaction was also significant, *F*(16, 1172) = 2.87, *p* < 0.001, η^2^_p_ = 0.04 (Fig. 1, Panel b). However, no differences emerged in the post-hoc comparisons. The post-hocs of the Condition x Letter and the Group x Letter interactions are graphically outlined in Figure S2, whereas additional analyses are reported in Text S3.

#### Homothety: cursive script

The GLM analysis on the relative letter duration revealed a significant Group x Letter interaction, *F*(16, 1172) = 4.54, *p* < 0.001, η^2^_p_ = 0.06 (Fig. 1, Panel d). The duration of the letter *b* in G1 was significantly shorter than the ones in G2, G3, and G4. The duration of letter *l* in G1 was significantly longer than those in G3, G4, and G5 (post-hoc tests are outlined in Figure S2). The interaction Condition x Letter was also significant, *F*(8, 2344) = 34.95, *p* < 0.001, η^2^_p_ = 0.11 (Fig. 1, Panel c). The relative duration of the letter *b* and *r* was shorter in the *Fast* condition as compared to the *Spontaneous* and *Big* conditions. The duration of the letter *u* was shorter in the *Fast* condition as compared to the *Spontaneous* condition. The post-hoc comparisons of the Group x Letter and the Condition x Letter interactions are graphically outlined in Figure S2, whereas additional analyses are reported in Text S4.

Similarly, to what was observed for the all-capital block script, no significant differences were found between the letters in the *Spontaneous* condition and the letters in the *Big* condition.

Overall, the results showed that children comply with Homothety since the first grade of primary school, both when writing in all-capital block script (Fig. 1, Panel b) and when writing in cursive script (Fig. 1, Panel d). A minimal deviation from Homothety is found in G1 children only for the letter *b* and *l*. However, this explains only the 5% of the variability.

Moreover, the results show that the rhythm of the word is generally preserved across conditions, as the relative durations of the single letters are very similar despite major changes in size and speed (see Fig. 1). In fact, the relative durations are kept substantially invariant across changes in size, as no significant differences emerged between the *Spontaneous* and *Big* conditions, both when writing in all-capital block script and in cursive script. The effect of task condition on Homothety appears to be driven by the fact that the instruction to write *Fast* slightly modifies the relative duration of letters within a word. Notably, this does not occur in the *Big* condition, where handwriting velocity is comparable to the *Fast* condition (see Table S1), but is spontaneously achieved as a result of Isochrony. In other words, when task requirements force participants to change the size of their handwriting, Homothety is preserved. When task requirements force participants to change the speed of handwriting, Homothety is violated only to a limited extent.

### Isochrony

**Figure 2:**
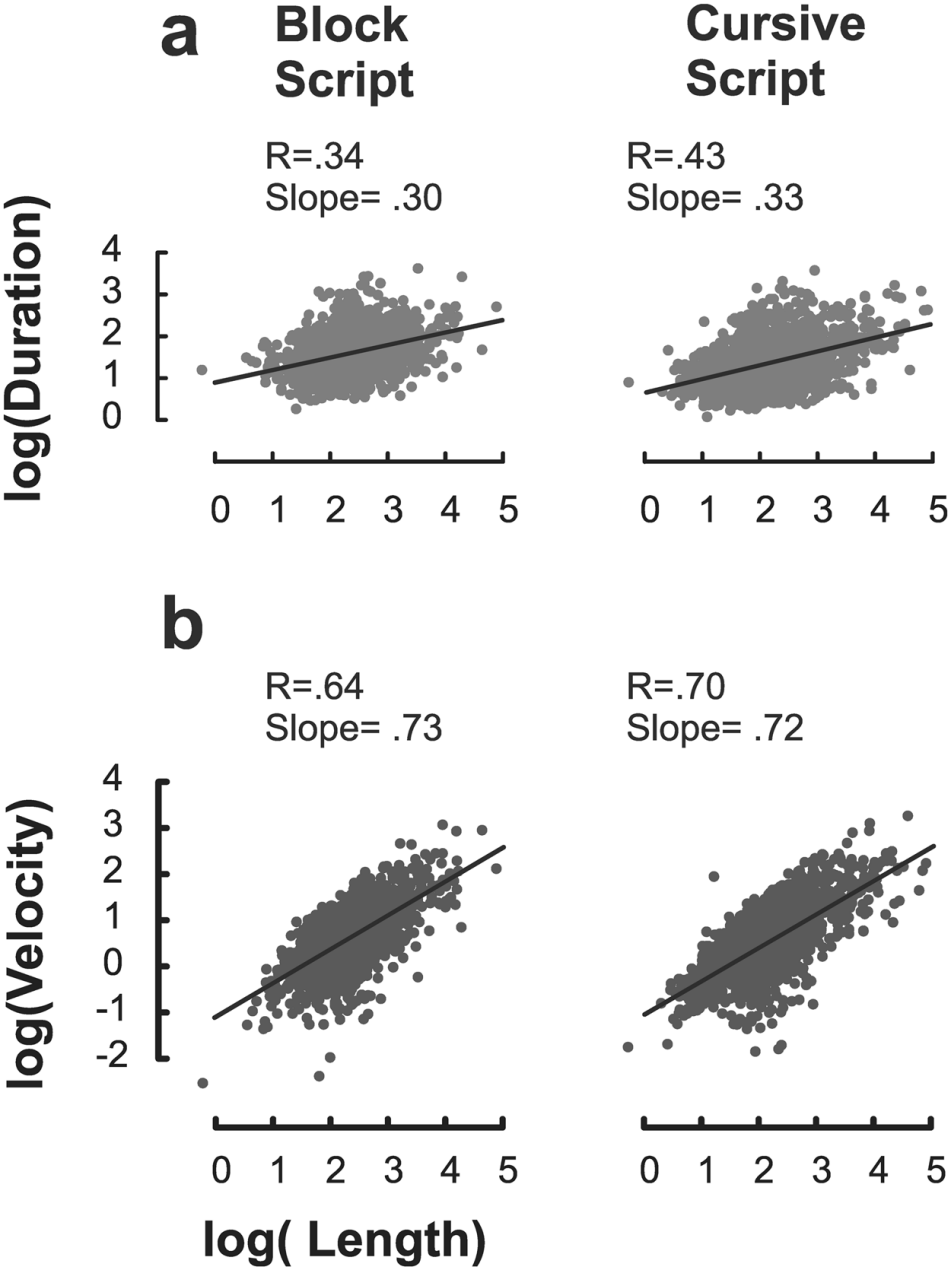
Isochrony. Panel a reports the log-log plot (natural logarithm) of the word duration (sec) as a function of its length(cm). Panel b reports the log-log plot (natural logarithm) of the mean velocity (cm/sec) as a function of its length. Subpanels on the left represent all-capital block script data, subpanels on the right depict cursive data. All log-log plots were computed on the entire dataset, thus including the five groups of participants all together and the conditions *Spontaneous*, *Big*, and *Fast*.

Figure 2, Panel a illustrates the word duration (sec) as function of its length (cm), as the log-log plot of the entire dataset (including the five groups of participants all together and the conditions *Spontaneous*, *Big*, and *Fast*). The left panel depicts all-capital block script data (intercept: 0.89, slope: 0.30, with r = 0.34, *p* < 0.001), whereas the right panel depicts the cursive script data (intercept: 0.65, slope: 0.33, with r = 0.43, *p* < 0.001). Figure 2, Panel b reports the log-log plot of the whole dataset of mean velocity (cm/sec) against length (cm). Again, the left panel represents all-capital block script data (intercept: −1.09, slope: 0.75, with r = 0.64, *p* < 0.001) and the right panel cursive script data (intercept: −0.97, slope: 0.72, with r = 0.70, *p* < 0.001).

In sum, the results showed that the velocity of the handwriting movement is proportionally related to the length of its trajectory in order to keep the movement duration relatively steady across changes in size. Thus, when participants are required to write bigger, they increase their writing speed in order to minimize changes in the global movement duration, in line with Isochrony.

**Figure 3:**
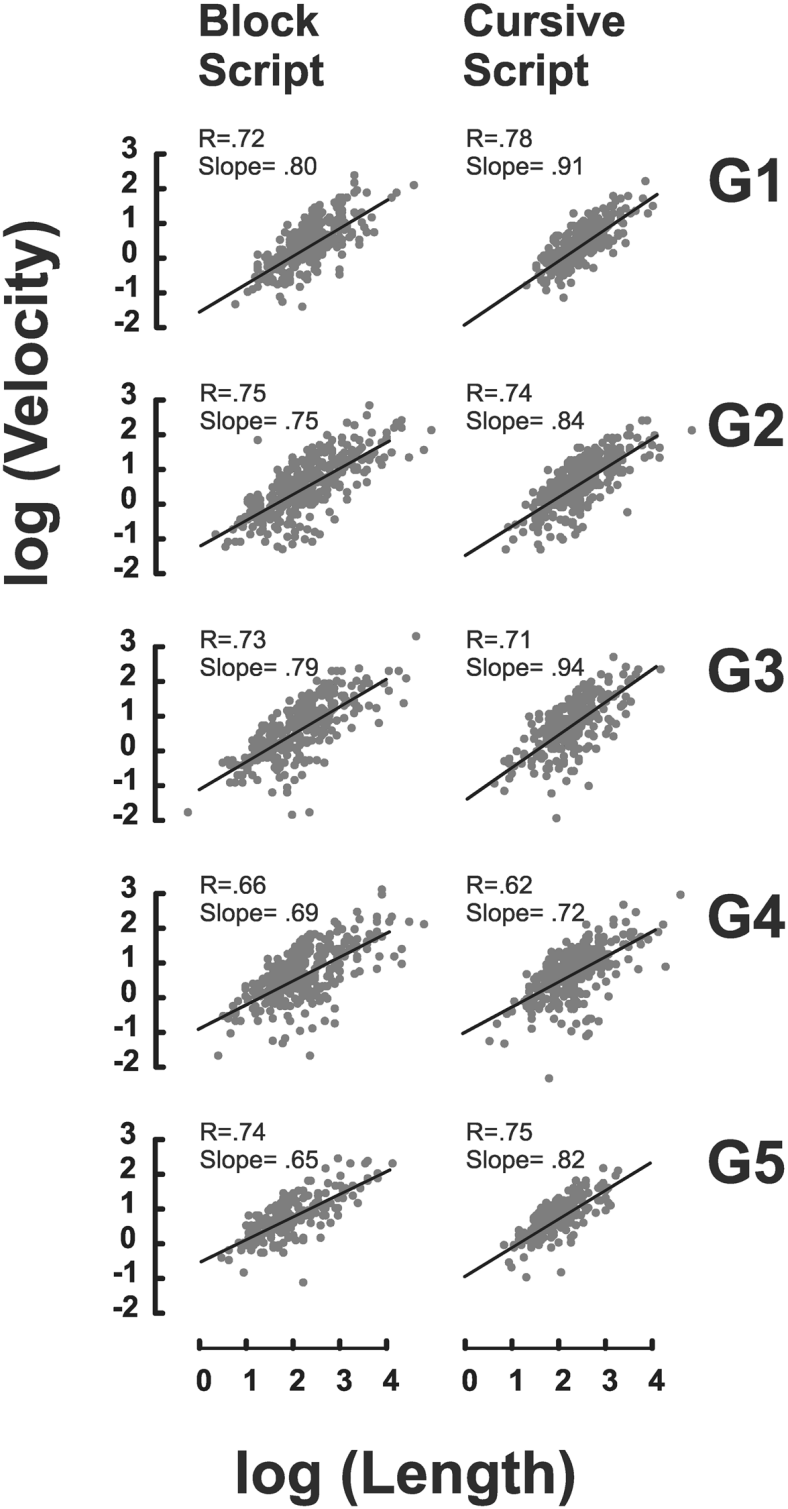
Isochrony is invariant across school grades. The log-log plot (natural logarithm) of length (cm) against mean velocity (cm/sec) is reported for each group of children - when writing in block and cursive scripts. Each data point represents the word *burle* written in one of the experimental conditions (*Spontaneous*, *Big*, and *Fast*) by each individual participant. In line with Isochrony, the mean velocity presents a strong positive correlation with the linear extent of the corresponding trajectory for each group of children.

Figure 3 reports the log-log plot of word mean velocity (cm/sec) against word length (cm) for each group when writing in block and cursive scripts.

The results at the group level showed that all the five groups of children equally adhered to Isochrony. In fact, each group presented a strong positive correlation between the word mean velocity and the linear extent of the corresponding trajectory. This holds both for the cursive and all-capital block scripts. All the pairwise comparisons between the correlation coefficients (r) were not significant (all-capital block script, *p* > 0.25; cursive script, *p* > 0.09). Thus, these results show that all groups of children from the first to the fifth grade of primary school complied equally well with the Isochrony principle.

## Discussion

In this study, we aimed at determining whether the principles that characterize rhythmic structure of handwriting, namely Homothety and Isochrony, are in place from the very first children’s productions.

With regards to Homothety, it is important to acknowledge that this is the first study that investigates this principle in a developmental perspective. Homothety^12,20^ dictates that the relative durations of movement components are kept constant across changes in speed. The results of the analysis on the relative letter duration showed that children comply with Homothety from the first grade of primary school, both when writing in all-capital block script (Fig. 1, Panel b) and when writing in cursive script (Fig. 1, Panel d). A very limited developmental effect was found for G1 in the cursive script (Fig. 1, Panel d). The perturbation of Homothety by G1 was very marginal and due to the letters *b* and *l* of the cursive script, whereas the relative durations of the letters *u*, *r* and *e* were comparable to those of the other groups. Thus, all groups of children were able to keep the relative durations of individual letters constant despite the changes in size and speed, both when writing in cursive and in all-capital block script. This is graphically represented in Fig. 1, Panels b and d, in which the curves representing the performances of the different grades overlap very well, indicating that all groups of children adhere to the principle of Homothety, with minor variations for G1 in the cursive script. Remarkably, this is the first piece of evidence showing that children from the first grade of primary school are able to keep the temporal relationship of all the units composing a motor act invariant despite changes in size and tempo.

The analysis on the relative letter duration also revealed an interesting effect of task condition. It emerged that Homothety is violated, albeit only to a very limited extent, when the experimental instructions involve a direct modification of the speed of handwriting (*Fast* condition) (see Fig. 1 Panel a and c). However, it is preserved when the experimental instructions apply to the handwriting size (*Big* condition). Noteworthy, the behavioral outcome of both experimental instructions is very similar in terms of handwriting velocity (see Table S1, *Fast* and *Big* conditions). However, the change in speed is spontaneously achieved as a result of the change in letter size in the *Big* condition. That is, the change in size constrains spontaneously and automatically the speed of the handwriting movement (Isochrony), yet the opposite does not hold: the requirement of writing faster does not automatically imply a comparable change in size (see Table S1). This lack of modulation in size likely affects the geometry of handwriting (e.g., with some distortions in the individual shape of the letters), which in turn impacts the kinematics of the motor act and thus induces violations in the Homothety principle. This pattern of results can be explained in the light of the interpretation of the so-called “Two-thirds Power Law” proposed by Papaxanthis *et al*.^38^. The “Two-thirds Power Law”^20,21,39–41^ is a robust motor law which states that the velocity of a planar arm movement is modulated by the local geometry of its trajectory. In other words, the “Two-thirds Powel Law” predicts that the movement velocity systematically depends on the curvature of the trajectory, so that movement velocity decreases as the curvature increases. Papaxanthis *et al*.^38^ proposes that only the movement geometry is settled during the planning of the movement itself whereas its kinematics derives automatically from geometry planning. In contrast, the reverse does not hold and changes in the kinematics of the movement do not have an automatic effect on geometry. The asymmetry we found between the *Spontaneous* and the *Big* conditions on one side and the *Fast* condition on the other side is in agreement with this interpretation of the “Two-thirds Power Law”.

In regard to Isochrony^15,17,20^, the results of the analysis on the whole word showed that when children were required to write bigger, as in the case of the *Big* condition, they tended to increase the velocity in order to keep the absolute duration relatively steady, as predicted by the principle of Isochrony. This evidence already emerges from the preliminary analysis (Text S2), which also remarkably showed that the relationship between size and speed of movement is not bi-univocal. In fact, albeit children did write faster when asked to write bigger, the opposite was not observed: the requirement to write faster did not automatically involve an increase in word trace length.

The results of the regression analysis on the whole word showed that the mean velocity strongly covariates with the length. Moreover, changes in duration are minimized across changes in size (a twentyfold increase in word trace length is needed in order to obtain an increase of 2.5 times in word duration).

Remarkably, Isochrony holds for all the grades equally consistently and for both scripts, as clearly depicted in Fig. 3. In fact, the correlation between the word mean velocity and the linear extent of the corresponding trajectory was very strong and comparable for all groups of children, both when asked to write in capital block script and in cursive script. The result of the cursive script is particularly important if we consider that, at the time of the testing session, G1 children had at most few months of training in this script. Taken together, our findings show that Isochrony characterizes the handwriting movement from the first grade of primary school, when children are around 6 years old (G1 children had a mean age of 6;8 and an age range of 6;1–7;6 years) and remains stable throughout all the grades of primary school (see Text S5).

Therefore, these findings suggest that Isochrony might be an inherent aspect of motor planning during handwriting. Certainly, Isochrony does not depend on practice time nor requires years of practice in order to be implemented, at variance with other motor aspects of handwriting^4–6,8^. Our results are compatible with previous studies investigating the development of Isochrony in the context of drawing movements. These studies^28,31^ showed that children at the age of 5 complied with Isochrony when drawing circles and ellipses. However, we believe that our study brings about more compelling evidence in favor of the view of Isochrony as an inherent aspect of motor planning: at variance with drawing circles and ellipses, handwriting represents a very complex motor skill that was most unlikely to have been mastered and automatized at the time of testing of the youngest age group^5,8,42^.

If we consider the present findings on Isochrony and Homothety in the light of traditional research on the development of handwriting, our results suggest the existence of some mechanisms of handwriting related to its rhythmic dimension that are present before handwriting movements turn ballistic and automatized. Previous studies have shown that motor automation in handwriting is reached around the third grade of primary school (ages 8–9 years old typically)^4–6,8^. Remarkably, in the current study children complied with Homothety and Isochrony already in their first grade of school. On the basis of these results, it is plausible to argue that both the Homothety and the Isochrony principle are neither the outcome of training nor are they influenced by training time/length. Still, are these principles innate features of the motor planning system? The present work leaves this question open and an answer to it will require future research.

Nevertheless, the early adherence to these two mechanisms is particularly noteworthy if we consider recent results on typically developing children and children with Developmental Dyslexia of age 9^37^. Children with Developmental Dyslexia and children with Dyslexia plus Dysgraphia were tested by means of the same experimental design adopted in the present study. The results showed that in both cohorts of children with Dyslexia the principles of Isochrony and Homothety were consistently and substantially violated, at variance with the results obtained for typically developing children who complied well with both principles^37^. In the light of the present results, it seems unlikely that the lack of adherence to Isochrony and Homothety by children with Dyslexia is due to the fact that these children have less experience in handwriting than typically developing children. Rather, the findings discussed in the present paper suggest that children with Dyslexia suffer from a profound impairment in the motor planning of specific features related to the timing dimension.

In the present paper, we demonstrated that since the first months of primary school, handwriting cannot be accounted for as a mere sequence of distinct acts, rather as a hierarchical process where each motor component occupies a precise place in a larger processing unit – even before being completely automatized.

Handwriting appears to be characterized by an inherent rhythmic structure since the child’s earliest written outputs, and this rhythmic structure appears to be available to the child’s mental model before actual execution. Thus, our brain is equipped with exceptionally tuned writing mechanisms. One of those is anchored in specific motor programs prescribing the temporal dimension of the handwriting event. This suggest that, despite being a cultural invention, writing must have evolved within our brain’s circuits and certainly the way we write is constrained by our biology. The intimate link between culture and biological organization can be also interestingly noticed in recent evidence showing how handwriting changes profoundly our brain. In fact, recent behavioral studies showed that handwriting training, but not typing practice, improves recognition of new characters both in preliterate children^43^ and adults^44,45^ and that handwriting has a significant influence in learning to read^43–45^.

Therefore, the act of writing is so easily taken for granted that we forget what an astonishing accomplishment and elaborate process it is. The present data suggest that, despite being a cultural invention, handwriting fits perfectly the constraints of our biological architecture.

## Methods

### Participants

Two hundred ninety-eight Italian monolingual children were tested. Children were divided into five groups according to their school grade: a group of first grade children - henceforth G1 (*N* = 57); a group of second grade children - henceforth G2 (*N* = 72); a group of third grade children - henceforth G3 (*N* = 61); a group of fourth grade children - henceforth G4 (*N* = 68); and a group of fifth grade children - henceforth G5 (*N* = 40). Children in each school grade group were approximately evenly divided as to gender. Demographic information of the participants is shown in Table S2. Children were recruited from three different schools in the provinces of Milan and Pavia (Italy) in order to reduce possible effects of a specific teaching and training method (Text S6).

The tenets of the Declaration of Helsinki^46^ were observed and the study was approved by the Ethics Committee of the University of Milano-Bicocca (nr. II-18).

Participants’ parents were fully informed about the study and signed informed consent before the testing session.

### Experimental design

Participants were asked to manipulate their handwriting movement velocity and size. The Italian word *burle* (English translation: “jokes”) was chosen as the target word because it is usually written in a smooth, continuous line when writing in cursive script.

Two pairs of contrasting conditions (*Big* versus *Small*; *Fast* versus *Slow*) were included in the experimental design in order to foster a natural change in size and velocity of the handwriting according to the size and the speed of the experimental conditions. The same task was conducted both in all-capital block letters script and in cursive script, and the order of execution of the script was fixed for all participants: first all-capitals block script and then cursive script. For each script, children were first asked to write the target word in the *Spontaneous* condition, i.e., as they habitually do in class, with no further instruction. The *Spontaneous* condition served as a baseline. Shortly after, children were asked to write the same word in other four different conditions: smaller (*Small* condition), bigger (*Big* condition), slower (*Slow* condition), and faster (*Fast* condition) with respect to the *Spontaneous* condition. Thus, the word *burle* was written ten times in total (Figure S1) varying in script, size, and speed. This experimental method has been adopted in previous studies to assess participants’ ability to control handwriting size and tempo, with target words of different languages (French: refs^47,48^; Italian: ref.^37^).

### Procedure

Each participant was tested individually in a quiet room at her/his school. The whole testing session lasted approximately 15 minutes. Before testing, the experimenter demonstrated to each child the use of the digitizing tablet: she showed that the pen functioned as a normal pen and that the tablet recorded the handwriting movement on-line. In order to foster modulation, participants were not given any template.

### Apparatus

Children were asked to write on an unruled A4 size paper rested in landscape orientation on the recording surface of an Intuos 3 Wacom tablet (Sampling rate: 200 Hz; physical size (W × D × H): 440 × 340 × 14 mm; active area (W × D): 305 × 231 mm; pressure sensitivity: 1.024; levels resolution: 5.080 lpi; pen accuracy: ±0.25 mm; tilt: ±60 degrees; maximum reading height with Pen: 6 mm). Children were required to grasp the wireless pen of the digitizing tablet with their dominant hand as if they were writing normally with a common pen. The digitizing pen left a visible ink trace on the paper and therefore the handwriting activity took place in a very realistic way. The digitizing tablet was connected to a computer via a USB cable. The data were acquired using VBDigitalDraw 2.0 software^49^. The VBDigitalDraw 2.0 Software permits the collection of an ample set of geometric, kinematic, and dynamic descriptors of handwriting. The same system has been recently employed to investigate the handwriting abilities of Italian dyslexic children with and without Dysgraphia^37^.

The trajectory of the handwriting was recorded as Cartesian coordinates (*x*, *y*), both when the pen tip was in contact with the surface of the digitizing table and when the pen tip position was in the air above the digitizer active area with pressure = 0, i.e., when the writer was temporary pausing or planning the next movement sequence.

The continuous string of the handwriting performance was segmented into single words, to investigate the principle of Isochrony, and letters, to study the principle of Homotethy. We started from an automatic segmentation procedure from the VBDigitalDraw 2.0 software. The automatic procedure segmented the handwriting trace on the basis of the detachment of the pen tip from the tablet surface, i.e., when pressure was equal to 0. Besides that, a temporal detachment greater than 5000 ms was considered the end of a segment.

After the end of a segment, the detection of a pressure greater than 0 was considered the beginning of a new segment. Segments shorter than 40 ms were considered artifacts and excluded from all subsequent analyses. The geometrical passage between two adjacent letters was stipulated at the minimum of velocity in the transition segments. The outputs of the software segmentation procedure were double-checked by two independent experimenters: the automatic segmentation into separate words was practically always correct. As for the automatic segmentation into single letters, the software outputs typically matched the standard geometry of letters in the all-capital block script very well. Instead, the cursive script sometimes required off-line manipulation, mostly due to the fact that cursive handwriting of the word *burle* is generally carried out without lifting the pen from the tablet. In case both independent experimenters identified a mismatch between the software output and the letter geometry, the letter segmentation was corrected manually by one of the experimenters as to match the standard letter geometry of the cursive script.

### Data analysis

To investigate the principle of Homothety, we examined the *relative letter duration*, i.e., the time required to write each letter of the word *burle* expressed as the ratio between the duration of the individual letter and the duration of the whole word (in percentage), separately for each participant and experimental condition.

For the investigation of the principle of Isochrony, we considered:

- *Word duration*: the time to write the whole word *burle* expressed in seconds, considering only the time during which the pen was in contact with the surface.
- *Word Length*: the sum of the length of all the strokes composing the word expressed in cm.
- *Word Mean velocity*: the mean velocity of pen movement expressed in cm/sec.

When asked to write in the *Small* condition, children tended to write extremely small. Similarly, when asked to write in the *Slow* condition, the majority of children tended to write very small. Therefore, we excluded the *Small* and the *Slow* conditions from the analyses as the data collected in these two conditions were not reliable for segmentation of letters due to the resolution limits of the digitizing tablet (±0.25 mm), by analogy with^37^. Even so, the inclusion of the *Small* and *Slow* conditions in the experimental design was necessary in order to bring about participants to modulate their handwriting size and speed.

### Statistical analyses

#### Homothethy

In order to investigate whether handwriting is governed by Homothety from the very first handwriting productions and preserved across ages, analyses were performed on letters as the selected segment. The relative duration of each letter was expressed as the average percentage of time the letter took to be written over the total duration of the word, separately for each participant and experimental condition.

Relative letter durations were analyzed using a Generalized Linear Model (GLM) analysis, with Group (G1, G2, G3, G4, G5) as a between-participant factor and Condition (*Spontaneous*, *Big*, *Fast*) and Letter (*b*, *u*, *r*, *l*, *e*) as within-participant factors.

Significant main effects and interactions were followed up using Bonferroni’s post-hoc comparisons. Significant values were reported (*p* < 0.05) for main effects and interactions. Partial eta squared (η^2^_p_) was reported as a measure of effect size.

As the main factors group and condition were converted to percentage (normalization), we could no longer evaluate their across-level change, and thus their main effect.

#### Isochrony

To investigate the principle of Isochrony, an analysis was performed with words as the selected target unit of analysis, with the data of all five groups of children and the conditions *Spontaneous*, *Big*, and *Fast* collapsed together. Linear regression analyses were used to examine the relationship between length and word duration and between length and mean velocity, all transformed in logarithmic values (natural logarithm). Five outlier values (4 referring to word duration and 1 referring to word length), all of which exceeded 4 logarithmic units above or below the expected value computed on the basis of the remaining 2980 pairs of values (log(length), (log(duration)), were substituted in the regression by the expected value itself.

We then run a separate regression for each group, including the conditions *Spontaneous*, *Big*, and *Fast* collapsed together.

### Data availability

The datasets analyzed during the current study are available at ﻿DOI: 10.20366/unimib/unidata/SN180-1.0.

## Electronic supplementary material

Supplementary material
