# Author Correction: Children’s first handwriting productions show a rhythmic structure

**DOI:** 10.1038/s41598-018-23228-2

**Published:** 2018-03-15

**Authors:** Elena Pagliarini, Lisa Scocchia, Mirta Vernice, Marina Zoppello, Umberto Balottin, Sana Bouamama, Maria Teresa Guasti, Natale Stucchi

**Affiliations:** 1 Center for Brain and Cognition (CBC), Departament de Tecnologies de la Informació i les Comunicacions (DTIC), Universitat Pompeu Fabra, c\Ramon Trias Fargas, 25-27, Barcelona, 08005 Spain; 2 Department of Psychology, Università degli Studi di Milano-Bicocca, Piazza dell’Ateneo Nuovo, 1, 20126 Milan, Italy; 3 Child Neuropsychiatry Unit, C. Mondino National Neurological Institute, Via Mondino, 2, 27100 Pavia, Italy; 4 Centre for Visual Cognition, School of Psychology, University of Southampton, Building 44R 4049. University Road, SO17 1BJ Southampton, UK; 5 Department of Brain and Behavioral Sciences, University of Pavia, Pavia, Italy

Correction to: *Scientific Reports* 10.1038/s41598-017-05105-6, published online 17 July 2017

The original version of this Article omitted an affiliation for Umberto Balottin. The correct affiliations are listed below:

Child Neuropsychiatry Unit, C. Mondino National Neurological Institute, Via Mondino, 2, 27100, Pavia, Italy.

Department of Brain and Behavioral Sciences, University of Pavia, Pavia, Italy.

This has now been corrected in the HTML and PDF versions of this Article, and the Supplementary Information file that accompanies the Article.

