# Supplementary material for: Children’s first handwriting productions show a rhythmic structure
Source: Sci Rep. 2017 Jul 17;7:5516. doi: 10.1038/s41598-017-05105-6 (PMC5514070; doi:10.1038/s41598-017-05105-6)
Supplement: Supplementary file 1 — Supplementary material [file 41598_2017_5105_MOESM1_ESM.docx]

**Children's first handwriting productions show a rhythmic structure**

**Authors (in order):** Elena Pagliarini^1,2,*^, Lisa Scocchia^2^, Mirta Vernice^2^, Marina Zoppello^3^, Umberto Balottin^3,4^, Sana Bouamama^5^, Maria Teresa Guasti^2^, Natale Stucchi^2^

**Author affiliation:**

^1^ Center for Brain and Cognition (CBC), Departament de Tecnologies de la Informació i les Comunicacions (DTIC), Universitat Pompeu Fabra, c\ Ramon Trias Fargas, 25-27, 08005, Barcelona, Spain

^2^ Department of Psychology, Università degli Studi di Milano-Bicocca, Piazza dell’Ateneo Nuovo, 1, 20126, Milan, Italy

^3^ Child Neuropsychiatry Unit, C. Mondino National Neurological Institute, Via Mondino, 2, 27100 Pavia, Italy

^4^ Department of Brain and Behavioral Sciences, University of Pavia, Italy

^5^ Centre for Visual Cognition, School of Psychology, University of Southampton, Building 44 R 4049. University Road. SO17 1BJ. Southampton, UK

***Corresponding author**

Elena Pagliarini

Center for Brain and Cognition (CBC)
Departament de Tecnologies de la Informació i les Comunicacions (DTIC)
Universitat Pompeu Fabra

c\ Ramon Trias Fargas, 25-27
08005 Barcelona
Edifici Mercè Rodoreda, 24.338

Phone number: +34 935 42 29 38

E-mail address: elena.pagliarini2@gmail.com

**SUPPLEMENTARY MATERIAL**

**Text**

**Text S1**

**Abjads**, or consonant alphabets, are writing systems consisting of consonants. Full vowel indication (vocalisation) can be added, usually by means of diacritics, but this is not usually done. Arabic and Hebrew are the most common examples of languages that use abjads^1^.

**Abugidas** are writing systems in which each consonant has an inherent vowel which can be changed to another vowel or muted by means of diacritics or other modifications. Vowels can also be written with separate graphemes when they occur at the beginning of a word or on their own^1^.

A **stroke** is a curve that can be traced without ever lifting or stopping the pen.

**Text S2**

**Preliminary analysis**

Descriptive data are reported in Table S1. Preliminary analyses were conducted in order to verify whether children had complied with the experimental requirements and had modulated their handwriting according to the task conditions.

**Table S1.** Mean (Standard Deviation in parentheses) word length (cm) and word mean velocity (cm/sec) of each experimental condition (5 groups collapsed).

| **Conditions** | **Word length**  **Block Script** | **Word length**  **Cursive Script** | **Word mean velocity**  **Block Script** | **Word mean velocity**  **Cursive Script** |
| --- | --- | --- | --- | --- |
| *Spontaneous*  *Big*  *Fast* | 9.62 (3.95)  26.85 (19.57)  10.33 (6.54) | 10.98 (4.33)  22.39 (14.83)  12.98 (7.35) | 1.95 (0.88)  3.92 (2.61)  3.94 (2.47) | 1.97 (0.83)  3.34 (2.11)  3.73 (2.24) |

We analyzed word length (cm) and word mean velocity (cm/sec) by means of Generalized Linear Model (GLM) analyses with group (G1, G2, G3, G4, G5) as a between-participants factor and condition (*Spontaneou*s, *Big*, *Fast*) as a within-participants factor. The significant comparisons reported in the text refer to Bonferroni post-hoc tests that meet at least the 5% significance level (*p* < .05). Partial eta squared (η^2^_p_) values were reported as a measure of effect size.

**Word length (cm).** With respect to all-capital block script, the GLM analysis on length revealed a main effect of group, *F*(4, 293) = 5.08, *p* < .001, η^2^_p_ = .06. Post-hoc comparisons showed that the length of the word written by G1 was shorter than that written by G2. The length of the word written by G5 was significantly shorter than those written by G2 and G4. Condition was also significant, *F*(2, 586) = 234.18, *p* < .001, η^2^_p_ = .44. Post-hoc comparisons showed that the length of the word written in the *Big* condition was longer than the length of the words written in the *Spontaneous* and *Fast* conditions and no difference was found between these two latter conditions. Finally, the analysis revealed a significant Group x Condition interaction, *F*(8, 586) = 4.04, *p* < .001, η^2^_p_ = .05. Post-hoc comparisons showed that, for all groups of children, the length of the word written in the *Big* condition was longer than those written in the *Spontaneous* and *Fast* conditions, with no difference between the latter two conditions. Thus, the significant interaction simply indicated a different modulation of the effect of condition across groups. Differences between conditions could be smaller or larger depending on group, still they can all be described in terms of the main effect of condition.

As for the cursive script, the GLM analysis on length (Group x Condition) revealed a main effect of group, *F*(4, 293) = 10.31, *p* < .001, η^2^_p_ = .12. Post-hoc tests revealed that the length of the word written by G5 was significantly shorter than the other groups. The length of the word written by G2 was significantly longer than all the other groups, except for G1. The length of the word written by G3 and G4 was shorter than that written by G2 but longer than that written by G5. The analysis also revealed a main effect of condition, *F*(2, 586) = 167.17, *p* < .001, η^2^_p_ = .36. Post-hoc comparisons showed that each condition differed from all other conditions, with the length of the word written in the *Big* condition being longer than the *Fast* and the *Spontaneous* conditions; the length of the word in this latter condition was the shortest. The analysis also showed a significant Group x Condition interaction, *F*(8, 586) = 3.48, *p* < .001, η^2^_p_ = .04. Post-hoc tests revealed that, for all groups, the length of the word written in the *Big* condition was significantly longer than the *Spontaneous* and *Fast* conditions, with no difference between the latter two conditions. The significant interaction is due to G2 and G4 children who wrote particularly big when asked to write bigger.

**Word mean velocity (cm/sec).** The GLM analysis of all-capital block script revealed a main effect of group, *F*(4, 293) = 3.86, *p* < .01, η^2^_p_ = .05. The target word was written slower by G1 as compared to G2, G3, and G4, whereas G1 did not differ from G5. Condition was also significant, *F*(2, 586) = 163.08, *p* < .001, η^2^_p_ = .36. Post-hoc tests showed that *burle* was written more slowly in the *Spontaneous* condition than in the *Big* and *Fast* conditions, with no difference between the latter two conditions. Finally, the analysis revealed a significant Group x Condition interaction, *F*(8, 586) = 3.05, *p* < .01, η^2^_p_ = .04. Post-hoc tests showed that for all groups, the word in the *Spontaneous* condition was written more slowly than in the *Big* and *Fast* conditions, and no difference was found between the *Big* and *Fast* conditions. Thus, the significant interaction showed that differences between conditions could be larger or smaller depending on group, but can all be explained in terms of the main effect of condition.

As for the cursive script, the GLM analysis revealed a main effect of group, *F*(4, 293) = 7.58, *p* < .001, η^2^_p_ = .09. Post-hoc tests showed G1 children were significantly slower than G2, G3, and G4, though G1 children did not significantly differ from G5 children. Condition was also significant, *F*(2, 586) = 151.54, *p* < .001, η^2^_p_ = .34. Post-hoc comparisons showed that each condition differed from the others, such that the word written in the *Spontaneous* condition was the slowest and that written in the *Fast* condition was the fastest. Finally, a significant Group x Condition interaction was found, *F*(8, 586) = 4.04, *p* < .001, η^2^_p_ = .05. Post-hoc tests showed that G1, G2, G3, and G4 wrote more slowly when writing in the *Spontaneous* condition than when writing in the *Big* and *Fast* conditions. G5 children wrote more slowly when writing in the *Spontaneou*s condition than when writing in the *Big* condition, whereas the difference between *Spontaneous* and *Fast* conditions was marginal (*p* = .12). For all groups of children, no difference was found between the *Big* and *Fast* conditions.

The results of the preliminary analysis confirmed our expectations. The overall significant difference between the *Spontaneous* and *Big* conditions in the total length and the difference between the *Spontaneous* and *Fast* conditions in the mean velocity confirmed that children complied with the task requirements and adjusted the size and the speed of their writing in accordance with the different conditions.

In addition to confirming that children have complied with the experimental requirements, these preliminary results showed that not only did children write faster in the *Fast* condition, but also in the *Big* condition since their first year of school. This result holds both when writing in all-capital block script and in cursive script. Therefore, these preliminary results provide the first piece of evidence in favor to the adherence to the Isochrony principle. Children as young as 6 years and one month showed a natural tendency to increase movement speed when asked to write bigger than usual. The results also showed that this tendency is preserved through all primary school grades, up to 11;2 year-old children.

**Text S3**

**Additional results on Homothety: all-capital block script**

The GLM analysis showed a main effect of letter, *F*(4, 1172) = 2317.60, *p* < .001, η^2^_p_ = .89. Post-hoc comparisons showed that each letter differed from the others (mean duration of the letter *b* = 33%, SD = 5%; *u* = 14%, SD= 3%; *r* = 22%, SD = 4%; *l* = 11%, SD = 3%; *e* = 19%, SD = 4%).

**Text S4**

**Additional results on Homothety: cursive script**

The GLM analysis on the relative letter duration revealed a main effect of letter, *F*(4, 1172) = 1195.60, *p* < .001, η^2^_p_ = .8. Post-hoc comparisons showed that the duration of the letters *b* (mean duration = 34%; SD= 7%), *u* (mean duration = 19%; SD = 5%), and *e* (mean duration = 12%; SD = 4%) differed from each other and from the duration of all other letters. The letters *r* (mean duration = 17%; SD = 4%) and *l* (mean duration = 17%; SD = 4%) differed from all the other letters, though they did not differ from each other.

**Text S5**

**General comments**

Our results are in contrast with previous evidence^2^ claiming that children comply less well with Isochrony than adults in handwriting. However, this claim rests upon a study conducted on a very small sample of participants (only 10 children were tested, ranging in age from 9 to 15) and by means of an apparatus of data acquisition with lower accuracy and ecological validity than ours. The handwriting speed was measured by means of a typewriter ribbon placed underneath a sheet of paper on which the participant wrote down. The speed on the typewriter ribbon was determined by an electric marker, which wrote tenths of a second on it. Thus, all these things considered, the data at the basis of the claim that children do not modulate speed in accordance with script size seem to be unreliable.

**Text S6**

**Participants**

Demographic information of the participants is reported in Table S2.

**Table S2** Demographic information: age, gender, and hand dominance of the participants.

| **Group** | **G1**  **(*n* = 57)** | **G2**  **(*n* = 72)** | **G3**  **(*n* = 61)** | **G4**  **(*n* = 68)** | **G5**  **(*n* = 40)** |
| --- | --- | --- | --- | --- | --- |
| Mean age in years  (SD in brackets) | 6;8  (0.3) | 7;7  (0.3) | 8;6  (0.3) | 9;6  (0.4) | 10;7  (0.3) |
|  |  |  |  |  |  |
| Age range | 6;1–7;6 | 6;4–8;3 | 7;9–9;3 | 8;2–10;7 | 10–11;2 |
|  |  |  |  |  |  |
| Gender |  |  |  |  |  |
| Male | 28 | 40 | 34 | 36 | 19 |
| Female | 29 | 32 | 27 | 32 | 21 |
| Hand dominance |  |  |  |  |  |
| Left | 8 | 6 | 4 | 12 | 8 |

Children were recruited from three different schools in the provinces of Milan and Pavia (Italy) in order to limit possible effects due to a specific teaching and training method. In the Italian system, handwriting starts to be trained in the first grade of primary school, when children are around 6 years-old. The teaching of block script precedes the teaching of cursive script and the time of introduction of this latter script is not uniform throughout schools, as some schools start the training of the cursive script during the second semester of the first grade of primary school whereas others introduce it at the beginning of the second grade. Therefore, in order to have homogeneous cohorts in term of exposition to the cursive script, we selected schools that introduce the training of cursive script during the second semester of the first grade. Moreover, in order to further control the time of exposition to the cursive script, all participants were tested in the second semester of the Italian school year (from the end of January to May).

All participants had a nonverbal IQ score (Raven’s Coloured Progressive Matrices)^3^ equal or above the 25^th^ percentile. In addition, a prior consultation with the teachers determined that the pupils had no cognitive, neural, motor, writing, or language problems (either clinically diagnosed or observed by the teachers themselves).

**Figures**


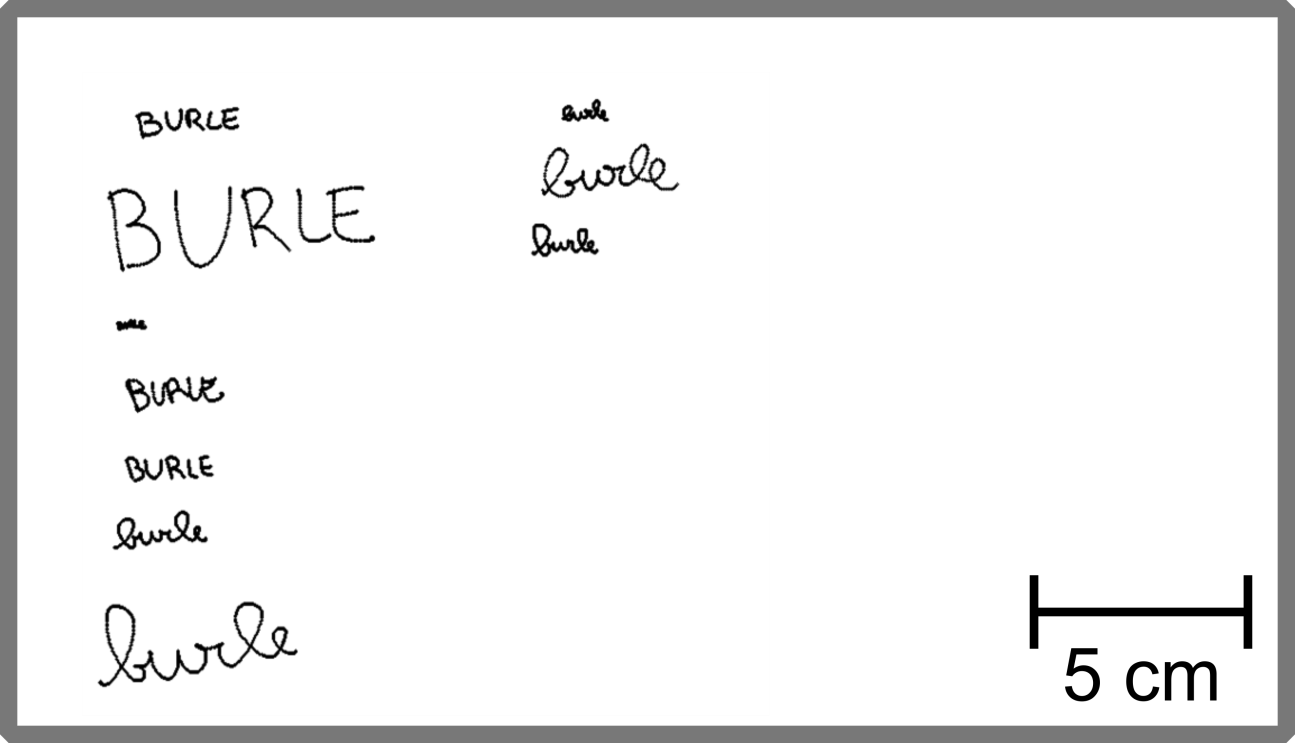


**Figure S1. Writing sample**. The writing output of a girl from G4 is shown. Children were asked to write the Italian word *burle* (English translation *jokes*) on an A4 landscape sheet of paper rested on the surface of an Intuos 3 Wacom tablet. Children were asked to write *burle* in five different conditions (*Spontaneous*, *Big*, *Small*, *Fast*, *Slow*) both in cursive and all-capital block script. The digitizing pen left a visible ink trace on the paper.

**
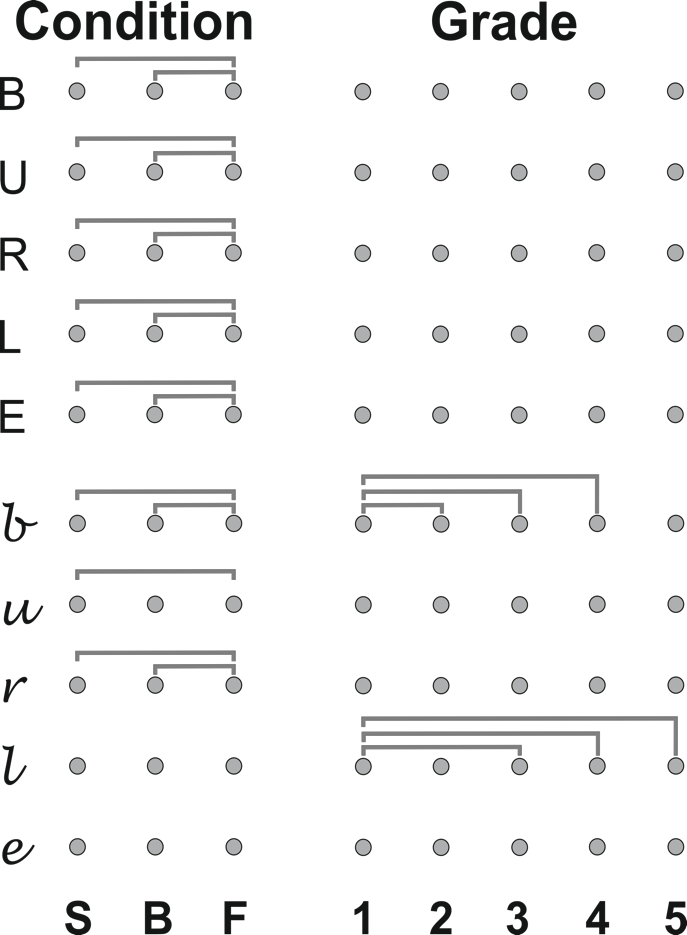
**

**Figure S2.** **Post-hoc comparisons**. The post-hoc comparisons of Condition x Letter and Group x Letter interactions are reported, both for block and cursive script. Significant differences are indicated by a bracket connecting two points, where each point represents a level of a factor. S stands for *Spontaneous*, B for *Big*, and F for *Fast*. 1 stands for G1, 2 for G2, 3 for G3, 4 for G4, and 5 for G5.

**References**

1. Ager, S. Omniglot: The Online Encyclopedia of Writing Systems & Languages. (1998).

2. Freeman, F. N. Experimental analysis of the writing movement. *Psychol. Monogr.* **17,** 1–57 (1914).

3. Raven, J. C., Court, J. H. & Raven, J. C. Coloured progressive matrices. (1998).
